# The amino acid sensor GCN2 suppresses terminal oligopyrimidine (TOP) mRNA translation *via* La-related protein 1 (LARP1)

**DOI:** 10.1016/j.jbc.2022.102277

**Published:** 2022-07-19

**Authors:** Zeenat Farooq, Fedho Kusuma, Phillip Burke, Catherine R. Dufour, Duckgue Lee, Negar Tabatabaei, Phoenix Toboz, Ernest Radovani, Jack F. Greenblatt, Jalees Rehman, Jacob Class, Arkady Khoutorsky, Bruno D. Fonseca, Justin M. Richner, Eloi Mercier, Guillaume Bourque, Vincent Giguère, Arvind R. Subramaniam, Jaeseok Han, Soroush Tahmasebi

**Affiliations:** 1 Department of Pharmacology and Regenerative Medicine, University of Illinois College of Medicine, Chicago, Illinois, USA; 2 Soonchunhyang Institute of Medi-bio Science (SIMS), Soonchunhyang University, Cheonan-si, Chungcheongnam-do, Korea; 3 Basic Sciences Division and Computational Biology Section of the Public Health Sciences Division, Fred Hutchinson Cancer Research Center, Seattle, Washington, USA; 4 Goodman Cancer Research Centre, McGill University, Montréal, Quebec, Canada; 5 Donnelly Centre, University of Toronto, Toronto, Ontario, Canada; 6 Department of Microbiology and Immunology, University of Illinois College of Medicine, Chicago, Illinois, USA; 7 Department of Anesthesia and Faculty of Dentistry, McGill University, Montreal, Quebec, Canada; 8 PrimerGen Ltd, Viseu, Portugal; 9 Canadian Centre for Computational Genomics, and Department of Human Genetics, McGill Genome Centre, McGill University and Genome Québec Innovation Center, Montréal, Quebec, Canada

**Keywords:** translational control, amino acid deprivation, GCN2, ATF4, GCN1, LARP1, TOP mRNA translation, ribosome collision, ACN, acetonitrile, ANS, anisomycin, BSA, bovine serum albumin, ChIP, chromatin-immunoprecipitation, CHX, cycloheximide, FA, formic acid, FBS, fetal bovine serum, FDR, false discovery rate, IP, immunoprecipitation, ISR, integrated stress response, KD, knockdown, MEF, mouse embryonic fibroblasts, MS, mass spectrometry, qPCR, quantitative PCR, RNP, ribonucleoprotein, RT-qPCR, reverse transcription qPCR, SG, stress granule, TOP, terminal oligopyrimidine, WB, Western blot

## Abstract

La-related protein 1 (LARP1) has been identified as a key translational inhibitor of terminal oligopyrimidine (TOP) mRNAs downstream of the nutrient sensing protein kinase complex, mTORC1. LARP1 exerts this inhibitory effect on TOP mRNA translation by binding to the mRNA cap and the adjacent 5′TOP motif, resulting in the displacement of the cap-binding protein eIF4E from TOP mRNAs. However, the involvement of additional signaling pathway in regulating LARP1-mediated inhibition of TOP mRNA translation is largely unexplored. In the present study, we identify a second nutrient sensing kinase GCN2 that converges on LARP1 to control TOP mRNA translation. Using chromatin-immunoprecipitation followed by massive parallel sequencing (ChIP-seq) analysis of activating transcription factor 4 (ATF4), an effector of GCN2 in nutrient stress conditions, in WT and GCN2 KO mouse embryonic fibroblasts, we determined that LARP1 is a GCN2-dependent transcriptional target of ATF4. Moreover, we identified GCN1, a GCN2 activator, participates in a complex with LARP1 on stalled ribosomes, suggesting a role for GCN1 in LARP1-mediated translation inhibition in response to ribosome stalling. Therefore, our data suggest that the GCN2 pathway controls LARP1 activity *via* two mechanisms: ATF4-dependent transcriptional induction of LARP1 mRNA and GCN1-mediated recruitment of LARP1 to stalled ribosomes.

It has been estimated that a single HeLa cell contains 3.3 × 10^6^ ribosomes, making ribosomes one of the most abundant macromolecular complexes in mammalian cells (1). Studies in yeast have demonstrated that about 60% of cellular energy is devoted to ribosome biosynthesis and ribosomal proteins account for 50% of the cellular proteome (2). To ensure availability of sufficient resources for ribosome biogenesis, cells have evolved sophisticated control mechanisms to coordinate the rate of ribosome production with nutrient and energy availability. mRNAs encoding ribosomal proteins (as well as a number of RNA-binding proteins and eukaryotic translation factors) carry a 5′-terminal oligopyrimidine (5′TOP) motif that is required for their translational control (3). The 5′TOP motif consists of a cytosine followed by an unbroken series of 4 to 14 pyrimidine nucleotides directly adjacent to cap structure (4, 5). The regulation of TOP mRNA translation by amino acid availability and other stressors has been recognized for many years and has been widely linked to the activity of mechanistic/mammalian target of rapamycin complex1 (mTORC1) (6, 7). However, the nature of downstream repressor proteins that interact with 5′TOP motif has been elusive until recently. Several proteins that mediate the inhibition of TOP mRNA translation have been proposed, and they include La antigen (8), AUF1 (9), 4E-BPs (10), TIA-1, and TIAR (11) as well as La-related protein 1 (LARP1) (4) which has been implicated in the process more recently. LARP1 is unique among other candidate repressors in that it physically interacts simultaneously with m7Gppp mRNA cap and the 5′TOP motif of TOP mRNAs *via* a specialized domain known as the DM15 domain (12, 13). LARP1 binds the mRNA cap of TOP mRNAs with higher affinity than a cap-binding protein eIF4E does (13). eIF4E, together with an mRNA helicase eIF4A and a large scaffolding protein eIF4G, forms eIF4F complex, which is essential for the initiation of cap-dependent translation. Therefore, the strong binding of LARP1 to the cap and TOP motif outcompetes eIF4E and consequently inhibits the assembly of the eIF4F complex, selectively blocking the translation initiation of TOP mRNAs (14). LARP1 was originally identified as a potential mTORC1 substrate in pharmacological phosphoproteomics studies (15, 16) and subsequently validated as a *bona fide* mTORC1 target (4). In response to a multitude of extracellular stimuli and intracellular cues such as growth factors and nutrients, mTORC1 phosphorylates LARP1 on multiple serine and threonine residues (17). Phosphorylation of LARP1 leads to the dissociation of the DM15 domain of LARP1 from the 5′UTR of TOP mRNAs, thus allowing eIF4F complex to access the 5′UTR of TOP mRNAs (13) and engage their translation (17). In addition to translational control, the activity of LARP1 has been linked to the sequestering of TOP mRNAs in stress granules (SGs) and P-bodies (18), preventing ribosome stalling (19) and regulating TOP mRNA stability (20, 21).

The serine/threonine protein kinase GCN2 (General control nonderepressible 2; also known as eIF2AK4) is an amino acid sensor that, similarly to mTORC1, coordinates the mRNA translation in response to amino acid availability. GCN2 is the most conserved member of the eIF2 alpha (eIF2α) kinases (eIF2AKs) in the integrated stress response (ISR) pathway that, as the name indicates, controls mRNA translation through phosphorylation of the alpha subunit of the eukaryotic translation initiation factor eIF2 (22). Phosphorylation of eIF2α on serine 51 blocks the guanine nucleotide exchange activity of eIF2B (the guanine nucleotide exchange factor [GEF] for eIF2), thereby hindering the eIF2/Met-tRNA_i_^Met^/GTP ternary complex formation. Ternary complex formation is required for the recruitment of small (40S) ribosomal subunit to mRNA, for translation initiation to take place (23). Since the ternary complex is essential for translation initiation of most cellular mRNAs, phosphorylation of eIF2α inhibits global mRNA translation. Paradoxically, phosphorylation of eIF2α stimulates the translation of a subset of mRNAs, which usually contain upstream ORFs in their 5′UTRs, epitomized by transcription factor activating transcription factor 4 (ATF4) mRNA (23). In turn, ATF4 orchestrates a transcriptional program that dictates how cells respond to stress (24). GCN2 contains a histidyl-tRNA synthetase (HisRS)-like domain that directly binds uncharged tRNA. Amino acid deprivation increases the level of uncharged tRNAs, which upon binding to GCN2 promote a conformational change within GCN2 that induces activation and autophosphorylation in *trans* (25, 26, 27). GCN2 can be also directly activated by the ribosome upon binding the ribosomal P-stalk *in vitro* or in the context of ribosome stalling (28, 29, 30, 31). GCN1 is required for full GCN2 activation and association of GCN2 with stalled ribosomes *in vivo* (32).

In addition to the effects of GCN2 on global translation and translation of mRNAs with upstream ORFs, the activity of GCN2 has been linked to translational control of specific TOP mRNAs (11) by an unknown mechanism. In this study, we demonstrate for the first time that GCN2 selectively suppresses TOP mRNA translation *via* transcriptional upregulation of LARP1 by ATF4. In addition, using immunoprecipitation in combination with mass spectrometry, we show GCN1 binds to LARP1 and colocalize with LARP1 on stalled ribosomes.

## Results

### GCN2-dependent ATF4 binding to the promoter of LARP1

**Figure 1:**
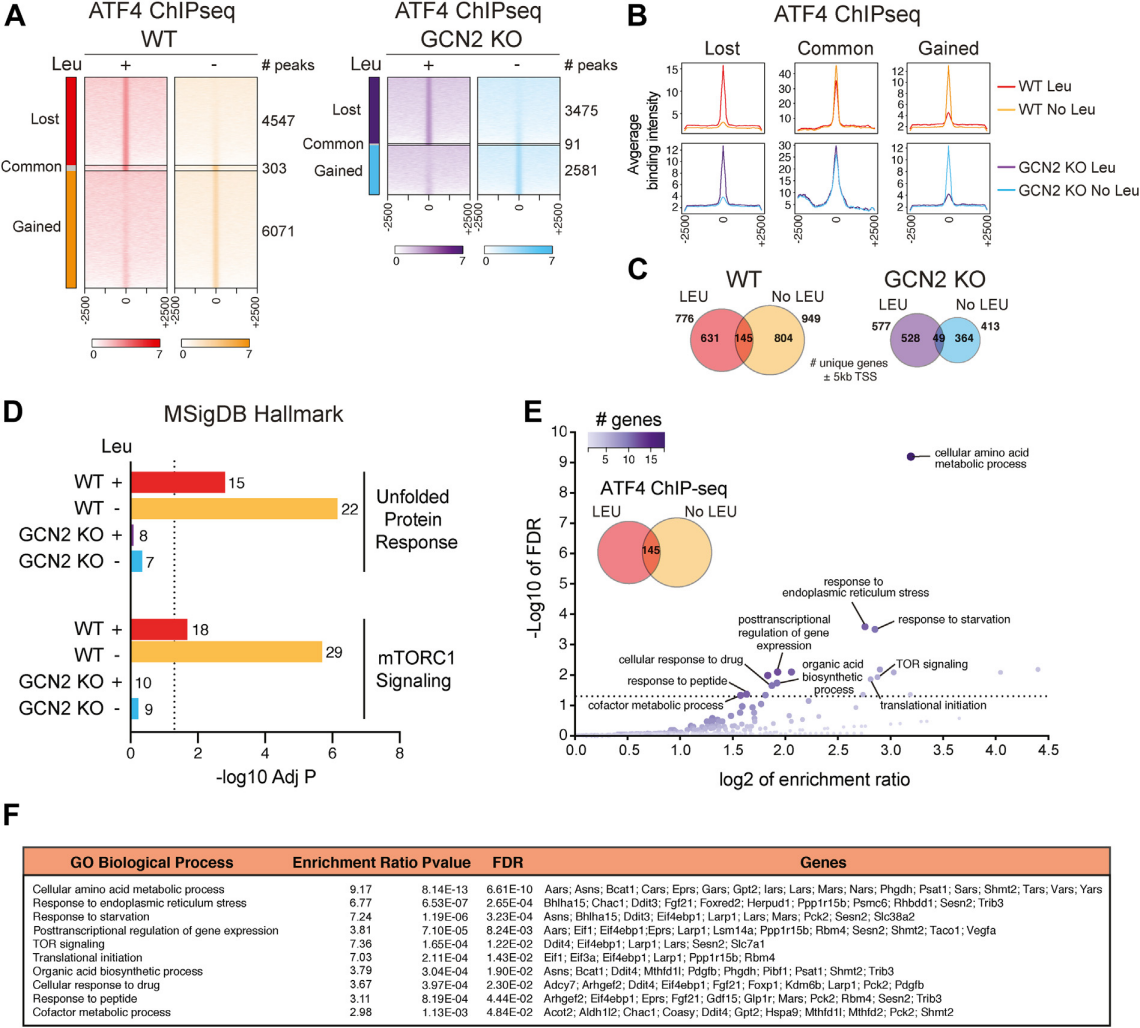
**ChIP-seq analysis of ATF4 in response to GCN2 activation.***A*, heatmaps of ATF4 ChIP-seq read densities in a window of ± 2.5kb from peak summits centered at 0 for WT and GCN2 KO MEFs exposed to control (+Leu) or leucine deficient (-Leu) medium for 24 h. Each row represents the ChIP-seq read density around a peak summit for each identified peak per condition. Read densities are proportional to color intensities across groups. ChIP-seq data represent a single sequencing experiment on a ChIP conducted using chromatin pooled from two independent experiments each performed with at least five replicates. In WT MEFs, leucine deprivation reprogrammed ATF4 binding and augmented the number of binding sites, an effect that is lost in GCN2 KO MEFs. *B*, average ATF4 ChIP-seq signal intensities from peaks identified in (*A*) normalized per reads for WT and GCN2 KO MEFs ± leucine (Leu) for 24 h. *C*, Venn diagrams illustrating the effect of leucine (Leu) deprivation on ATF4 ChIP-seq target gene identification in WT and GCN2 KO MEFs. The analysis was restricted to genes harboring peaks identified within ±5 kb of gene TSSs. *D*, enriched (adjusted *p*-value < 0.05) MSigDB Hallmark gene signatures in ATF4 ChIP-seq target gene sets with binding peaks found within ±5 kb of gene TSSs. *E* and *F* functional enrichment analysis of an ATF4-targeted 145-gene set with binding peaks present within ±5 kb of gene TSSs in WT MEFs ± leucine. Using a redundancy reduction of significant terms, the top 10 significantly enriched (Benjamin-Hochberg (BH)-corrected FDR < 0.05) GO biological processes determined by WebGestalt are shown with the associated genes. Node size and color are proportional to the number of genes found in a biological category. An enrichment ratio >1 denotes that the number of overlapping genes with a functional term is greater than by chance with a random set of genes. ChIP, chromatin-immunoprecipitation; FDR, false discovery rate; GO, Gene Ontology; MEF, mouse embryonic fibroblast; TSS, transcription start site.

**Figure 2:**
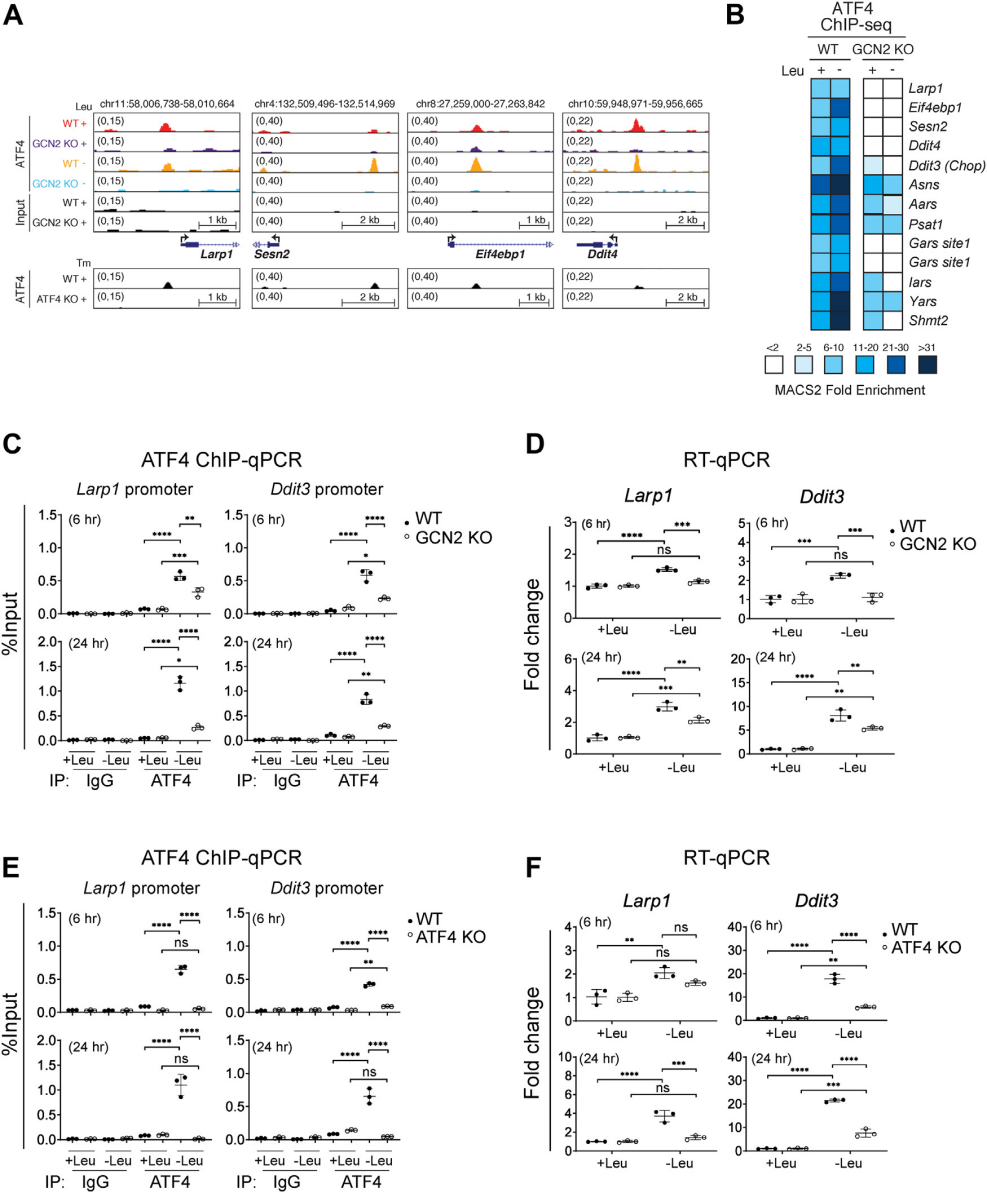
**GCN2-dependent regulation of the LARP1 *via* ATF4.***A*, UCSC Genome browser views for ATF4 ChIP-seq binding events in WT and GCN2 KO MEFs that have been exposed to control (+Leu) or leucine-deficient (-Leu) medium for 24 h for a subset of genes associated with mTOR signaling. ATF4 ChIP-seq binding profiles in WT and ATF4 KO MEFs treated with tunicamycin (Tm) (GSE35681) (41) are also shown below each panel validating the specificity of the ATF4 targets found. ChIP-seq data represent a single sequencing experiment on a ChIP conducted using chromatin pooled from two independent experiments, each performed with at least five replicates. *B*, a heatmap shows MACS2 fold enrichment for a selected ATF4 ChIP-seq target genes associated with mTOR signaling, the integrated stress response and amino acid metabolism with binding peaks found within ±5 kb of gene TSSs. ChIP-seq data represent a single sequencing experiment on a ChIP conducted using chromatin pooled from two independent experiments each performed with at least five replicates. *C* and *E*, ATF4 ChIP-qPCR analysis of *Larp1* and *Ddit3* promoters in WT and GCN2 KO MEFs (*C*) or WT and ATF4 KO MEFs (*E*) exposed to control (+Leu) or leucine-deficient (-Leu) medium for 6 h and 24 h. *D* and *F*, RT-qPCR analysis of *Larp1* and *Ddit3* in WT and GCN2 KO MEFs (*D*) or WT and ATF4 KO MEFs (*F*) exposed to control (+Leu) or leucine-deficient (-Leu) medium for 6 h and 24 h. Data in (*C*–*F*) are presented as means ± SD (n = 3). ∗*p* < 0.05, ∗∗*p* < 0.01, ∗∗∗*p* < 0.001, ∗∗∗∗*p* < 0.0001; Two-way ANOVA followed by Bonferroni post hoc test. ChIP, chromatin-immunoprecipitation; MEF, mouse embryonic fibroblast.

Previous studies demonstrated that GCN2 suppresses mTORC1 upon amino acid deprivation *via* ATF4-dependent or ATF4-independent pathways (33, 34). Prolonged starvation of leucine (8–24 h) is required for GCN2 to sustain mTORC1 suppression *via* ATF4 targets (33). To identify novel factors that control mTORC1 pathway downstream of GCN2-ATF4, we performed chromatin-immunoprecipitation (ChIP) followed by massive parallel sequencing (ChIP-seq) in WT and GCN2 KO mouse embryonic fibroblasts (MEFs) in the presence or absence of leucine for 24 h (Figs. 1, *A* and *B* and S1, *A* and *B*). Leucine deprivation induced a significant increase and reprogramming of ATF4-binding sites, an effect that was abolished in GCN2 KO cells (Fig. 1, *A* and *C*). A large number of known ATF4 targets was identified in our analysis including *Chac1* (35), *Kdm6b*/JMJD3 (36), *Asns* (37), *Arl14ep* (38), *ATF5* (39) and *Ppp1r15a*/*GADD34* (40), thus validating the quality of the ChIP-seq data. Interestingly, several important genes related to mTOR signaling (*e.g.*, *Ddit4/REDD1*, *Eif4ebp1*, and *Sesn2*), amino acid metabolism (*e.g.*, *Gars*, *Iars*, *Yars*, *Asns*, and *Psat1*), and integrated stress response (*Bhlha15/MIST1*, *Chac1*, *Ddit3/CHOP*, *Ppp1r15a/GADD34*, and *Ppp1r15b/CReP*) were prebound by ATF4 under basal conditions (Figs. 1, *D*–*F*, 2*A* and S1, *C*–*E*). ATF4 binding to these targets was largely stimulated by leucine deprivation, an effect that was blunted by loss of GCN2 (Figs. 1, *C* and *D*, 2, *A* and *B*). Among the novel ATF4 targets identified, we noted *Larp1* (Fig. 1*F*), a recently identified downstream effector of mTORC1 and a central regulator of TOP mRNA translation.

### ATF4 induces *Larp1* expression in response to amino acid deprivation

Similar to other genes related to mTOR signaling (*e.g.*, *Ddit4/REDD1*, *Eif4ebp1*, and *Sesn2*), *Larp1* promoter was prebound by ATF4 under basal conditions in WT cells, which overlapped with ATF4-binding locations previously identified in response to tunicamycin (Tm) treatment (41) (Figs. 2*A* and S1*D*). However, we did not observe a significant increase in ATF4 recruitment to the *Larp1* promoter after a 24 h leucine deprivation in ChIP-seq analysis (Fig. 2, *A* and *B*). Interestingly, ChIP-quantitative PCR (qPCR) analysis of *Larp1* indicates that the ATF4 binding to LARP1 promoter is induced as early as 6 h post leucine deprivation in WT MEFs, an effect that was largely blunted in GCN2 KO MEFs (Fig. 2*C*). Concurrently, LARP1 mRNAs were induced 6 h after leucine deprivation (Fig. 2*D*). Importantly, Western blot (WB) analysis confirmed higher LARP1 expression in WT compared to GCN2 KO MEFs at baseline and following L-leucine deprivation (Fig. S2*A*). To directly assess the central role of ATF4 in regulation of *Larp1*, WT and ATF4 KO MEFs were starved in leucine-free medium for 6 to 24 h (Fig. 2, *E* and *F*). We observed 6 h and 24 h leucine deprivation markedly increased ATF4 binding to the *Larp1* promoter, effects that were abolished in ATF4 KO MEFs (Fig. 2*E*). Reverse transcription (RT)-qPCR also showed marked increase in expression of *Larp1*, 6 h and 24 h after leucine deprivation (Fig. 2*F*).

### GCN2 inhibits TOP mRNA translation in response to amino acid deprivation

**Figure 3:**
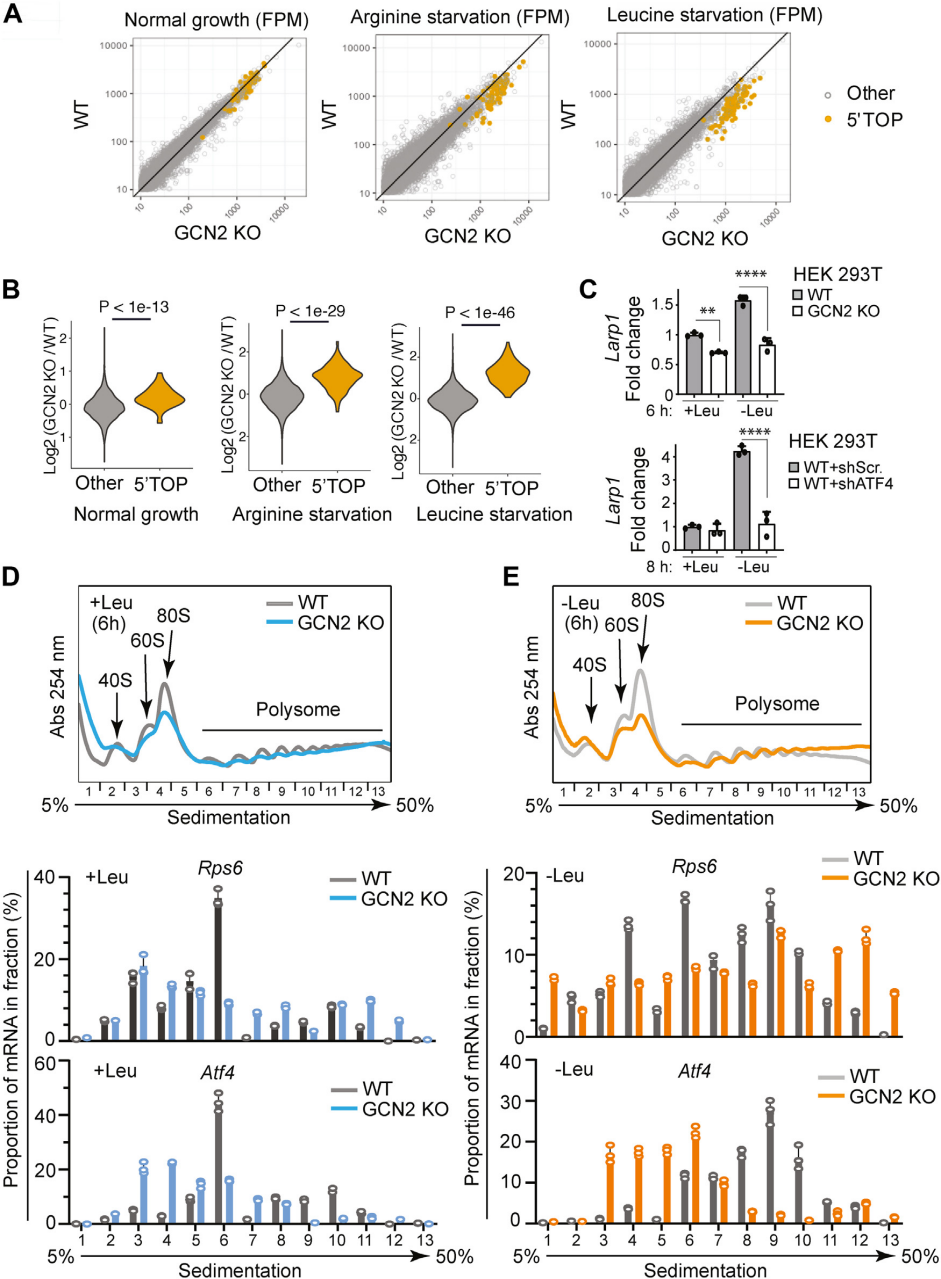
**GCN2 suppresses TOP mRNA translation.***A*, analysis of ribosome profilings performed in a previous study (Darnell 2018) on WT and GCN2 KO HEK293T cells after 6 h of arginine or leucine starvation or nutrient replete conditions (n = 1). Scatter plots of footprints per million (FPM) for each transcript are plotted for WT and GCN2 KO cells ± arginine or leucine starvation. *Yellow* points indicate 5′TOP mRNAs (the list of 5′TOP mRNAs obtained from Table S2 of Yamashita *et al*. 2008) (66). *B*, violin plots of Log2 (GCN2 KO/WT) values for all transcripts in each nutrient condition described in (*A*). A two-sided Wilcoxon signed-rank test was performed to assess whether the Log2 (GCN2 KO/WT) values of 5′TOP mRNAs increased significantly compared to non-5′TOP mRNAs. Resulting *p*-values are shown for each condition. *C*, RT-qPCR analysis of *LARP1* expression in WT and GCN2 KO HEK 293T cells or in HEK293T cells exposed to scrambled shRNA (Scr.) or shRNA against *ATF4* (shATF4) in the presence or absence of leucine (Leu). Data are presented as means ± SD (n = 3). ∗ *p* < 0.05, ∗∗ *p* < 0.01, and ∗∗∗ *p* < 0.001; Two-way ANOVA followed by Bonferroni post hoc test. *D*, absorption profiles of ribosomes and RT-qPCR analysis of WT and GCN2 KO HEK 293T cells cultured in control medium (+Leu) for 6 h. 40S and 60S denote the corresponding ribosomal subunits and 80S refers to monosomes. *E*, absorption profiles of ribosomes and RT-qPCR analysis of WT and GCN2 KO HEK293T cells cultured in the absence of leucine (-Leu) for 6 h. 40S and 60S denote the corresponding ribosomal subunits and 80S refers to monosomes. RT-qPCR, reverse transcription quantitative PCR; TOP, terminal oligopyrimidine.

Global analysis of translation targets of LARP1 demonstrated that LARP1 is a central inhibitor of TOP mRNA translation (42). Thus, GCN2-dependent regulation of *Larp1* predicts that activation of GCN2 in response to amino acid deprivation selectively suppresses TOP mRNA translation. Indeed, analyzing our ribosome profiling data in WT and GCN2-deficient HEK293T cells (43) demonstrates that lack of GCN2 selectively derepressed translation of most TOP mRNAs in response to both arginine and leucine deprivation (Fig. 3, *A* and *B*). GCN2-dependent translation inhibitions were not uniform across all TOP mRNAs, but instead, displayed an amino acid–specific sensitivity gradient (Fig. S2*B*). Consistent with MEFs, we observed GCN2-ATF4–dependent transcriptional regulation of LARP1 in HEK293T cells (Fig. 3*C*).

To directly examine whether GCN2 suppresses translation of TOP mRNAs, we performed a polysome profiling of WT and GCN2 KO HEK293T cells cultured in the presence or absence of leucine for 6 h. The absorbance profiles of the gradient fractions demonstrated an elevation in global translation in GCN2 KO cells compared to WT cells, both in presence and absence of leucine as indicated by higher 80S peak in WT cells (Fig. 3, *D* and *E*). These results were further verified by puromycin incorporation assay (Fig. S3, *A* and *B*). RT-qPCR analysis of mRNAs encoding the ribosomal proteins RPS6 and RPS20 (representative of TOP mRNAs in our analysis) showed their preferential association with heavier polysomes in the GCN2 KO HEK293T cells in comparison to WT cells (Figs. 3, *D* and *E* and S3*C*). Consistent with GCN2-dependent translational upregulation of ATF4, amino acid deprivation opposite to its effect on TOP mRNAs, shifted *Atf4* mRNAs toward heavy polysome more prominently in WT cells compared to GCN2 KO cells (Fig. 3, *D* and *E*). We next examined the effect of LARP1 knockdown (KD) on polysome distribution of TOP mRNAs (Fig. S3, *D*–*F*). Consistent with our model, LARP1 depletion profoundly alleviated the inhibition of TOP mRNAs in WT cells as judged by increased association of RPS6 and RPS20 with heavier polysomes (H) in LARP1 KO cells (WT-shLARP1) in comparison to control cells (WT-shScr.) (Fig. S3*E*). KD of LARP1 in GCN2 KO cells also further promoted the association of RPS6 to heavy (H) and RPS20 to light (L) polysome, indicating the functional significance of remaining LARP1 (Fig. S3*F*). Altogether, these results suggest that GCN2 controls the translation of TOP mRNAs through regulation of *LARP1* expression.

### GCN1 participates in a complex with LARP1 at stalled ribosomes

**Figure 4:**
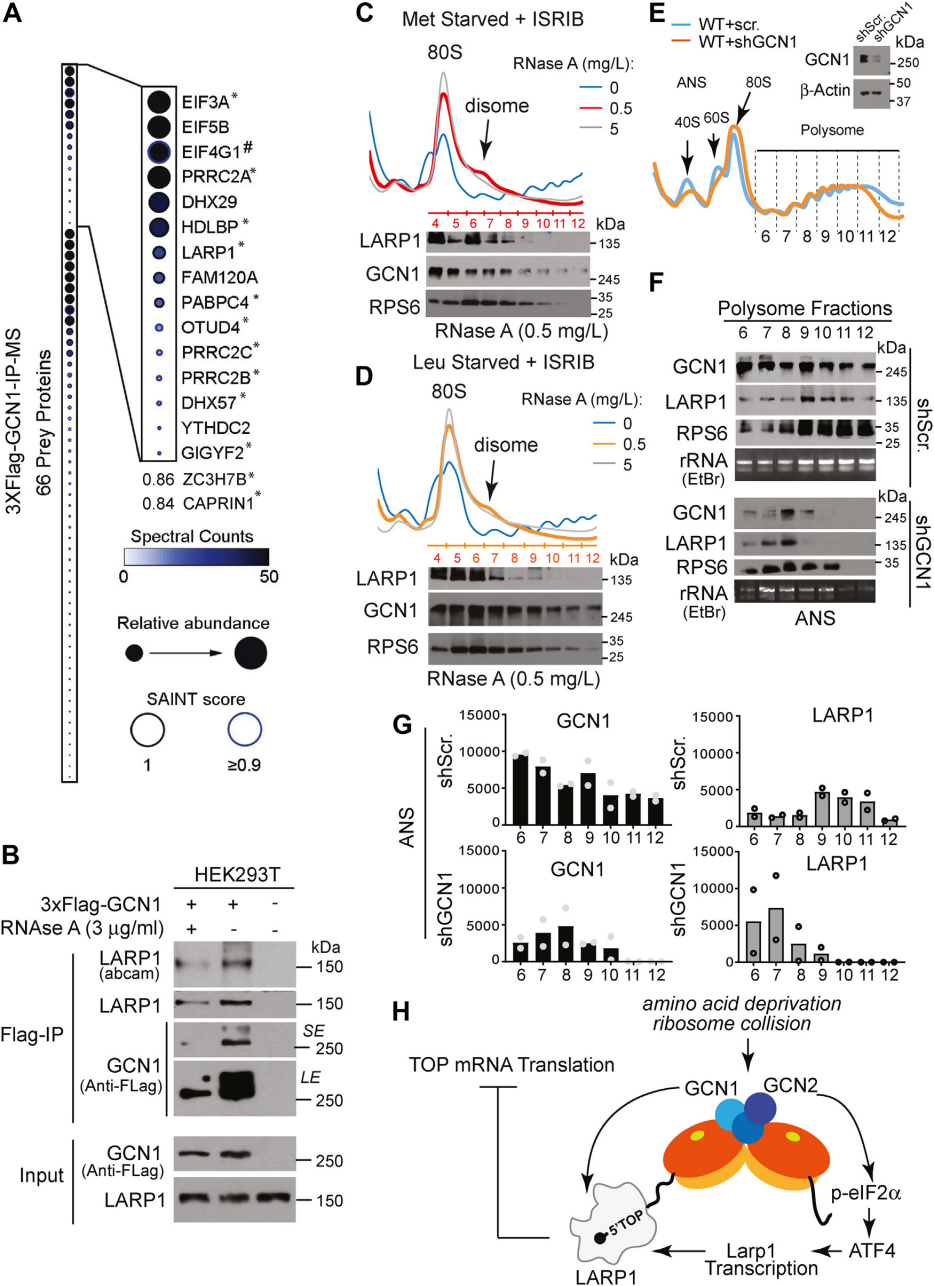
**GCN1 participates in a complex with LARP1atstalled ribosomes.***A*, mass spectrometric analysis of immunoprecipitates prepared from HEK293T cells endogenously expressing 3xFlag-tagged GCN1 (n = 1) identified LARP1 along with core components of PB (P-body) or SG (stress granule) proteomes (Youn *et al* 2009) (see Table S2 for complete list of the identified proteins).∗ Common preys from ZAKa, GCN1, and GCN20 Bio-ID identified in44. # EIF4G1 is exclusive to SGs. *B*, immunoblots of immunoprecipitates prepared from HEK293T cells endogenously expressing 3xFlag-tagged GCN1 in the presence or absence of RNAse A. SE; Short exposure, LE; Long exposure. *C* and *D*, amino acid starvation induces ribosome collisions and comigration of LARP1 and GCN1 toward disomes. RNase-digested polysome profiles from HEK293T cells starved for methionine (Met) (*C*) or leucine (Leu) (*D*) with ISRIB pretreatment. Immunoblot analysis of fractions of samples treated with 0.5 mg/L of RNase A has been presented below profiles. *E* and *F*, polysome profile (*E*) and immunoblot analysis (*F*) of WT HEK293T cells in the presence of scrambled shRNA (shScr.) or shRNA against GCN1 (shGCN1). The shScr. and shGCN1 cells were exposed to anisomycin (ANS) at 1 μg/ml for 15 min and cytosol was fractionated with sucrose density gradients. Precipitated protein from the polysome fractions (fractions 6–12) were examined by Western blotting (WB). *G*, quantification of WB described in (*C*) in two independent experiments (n = 2). *H*, a proposed model illustrating GCN2-dependent regulation of TOP mRNAs *via* LARP1. TOP, terminal oligopyrimidine.

Recent studies have shown ribosome collision during translation triggers the GCN2 pathway (44). Based on this model, in response to general cellular stress such as amino acid deprivation, elongating ribosomes stall, leading to collision of leading and trailing ribosomes. The resulting disomes are recognized by GCN1, which in complex with GCN2 and GCN20 activates the ISR pathway (31, 32, 45, 46). Some evidence suggests that LARP1 may play a role in resolving ribosome stalling during elongation of TOP mRNAs (19). Since LARP1 has been identified to colocalize with GCN1 (44, 47), we wondered whether GCN1 in response to ribosome stalling recruits LARP1 to block ribosome loading on TOP mRNAs. To test this hypothesis, we generated 3xFlag GCN1 knock-in HEK293T cells and performed immunoprecipitation (IP)–mass spectrometry (MS) to identify potential interaction between LARP1 and GCN1 under steady state (Fig. 4*A*). Indeed, LARP1 was among the most enriched prey proteins identified in our analysis. We directly validated the interaction of LARP1 and GCN1 by immunoblot analysis following 3xFlag GCN1 IP (using M2-agarose beads) (Fig. 4*B*). Removal of RNase A from the cell lysis buffer enhanced the signal intensity, indicating that the interaction between GCN1 and LARP1 is stabilized by RNA (Fig. 4*B*). Among 66 preys in the GCN1 purification (SAINT score of ≥0.9), several proteins have been previously identified to colocalize with GCN1 and LARP1 in BioID analysis (47) and have been implicated in activation of GCN2 in response to ribosome collision (44). In addition, some preys play a critical role in regulation of ribosome stalling (*e.g.*, USP9X (48) and GYGYF2 (49)). To assess the colocalization of LARP1 and GCN1 at stalled ribosomes, we pretreated HEK293T cells with ISRIB (to block inhibition of translation initiation) and exposed the cells to amino acid–deprived media for 1 h (44). As previously reported (44), RNase-resistant disomes appeared at a low RNase A concentration (0.5 mg/L), which promoted comigration of GCN1 and LARP1 toward disomes (Fig. 4, *C* and *D*). However, ribosome pausing secondary to amino acid deprivation is modest in comparison to that seen in response to treatment with translation elongation inhibitors such as anisomycin (ANS). Next, we treated 3xFlag GCN1 knock-in HEK293T cells with ANS at a concentration known to induce ribosome stalling (44) (Fig. S4*A*). Consistent with LARP1 recruitment to collided ribosomes, ANS treatment promotes comigration of GCN1 and LARP1 toward heavy polysome and the interaction between GCN1 and LARP1 (Fig. S4, *B*–*D*). Importantly, GCN1 KD dampened LARP1 migration to heavy polysome (Fig. 4, *E*–*G*). Therefore, we concluded that there are at least two mechanisms that GCN2 uses to inhibit TOP mRNA translation. In response to amino acid deprivation, GCN2 induces ATF4-dependent transcription of *Larp1*, and in response to ribosome collision (which may also be caused by amino acid deprivation), GCN1 recruits LARP1 to reduce loading of ribosomes on TOP mRNAs (Fig. 4*H*).

## Discussion

Our study reveals a novel link between the amino acid sensor GCN2 and the regulation of TOP mRNA translation through modulation of LARP1 mRNA, protein levels, and association with stalled ribosomes. Previous studies have demonstrated that lack of GCN2 promotes translation of specific TOP mRNAs through a yet unidentified mechanism (50). While Damgaard *et al.* proposed that GCN2 regulates translation of TOP mRNAs *via* SG-associated TIA-1 and TIAR proteins (11), other studies proposed alternative mechanisms such as a GCN2-eIF2α-dependent (51), GCN2-dependent eIF2α-independent (52), or indirectly through regulation of the mTORC1 pathway (34, 53). Our work demonstrates that there are at least two independent mechanisms that GCN2 uses to inhibit TOP mRNA translation *via* LARP1: (1) induction of ATF4-dependent transcription of LARP1 and (2) recruitment of LARP1 to stalled ribosomes through interaction with GCN1. Regulation of LARP1 by the GCN2 pathway complements the control of LARP1 activity by the mTORC1 pathway. The GCN2-ATF4-LARP1 axis likely represents a more sustained response to nutrient deprivation than the rapid but transient mTORC1 phosphorylation-mediated regulation of LARP1. This is reminiscent of coregulation of another cap-dependent translational repressor 4E-BP1 by mTORC1 and the ISR pathway, where transcriptional regulation of *eIF4E-BP1* (encoding 4E-BP1) by ATF4 complements phosphorylation-mediated regulation of 4E-BP1 by mTORC1 (54).

The present study reveals that GCN1 participates in a complex with LARP1 that strengthened by RNA and that this interaction is enhanced in response to ribosome pausing. Informed by a recent structure of the GCN1-disome (32), we built a model in which in response to amino acid deprivation and/or ribosome collision, GCN2 suppresses TOP mRNA translation through transcriptional control of LARP1 and recruitment of LARP1 protein to the site of ribosome collision (Fig. 4*H*). This model is consistent with other studies that demonstrated the recruitment of translation initiation repressors GIGYF2/4EHP by ZNF598 or EDF1 (direct sensors of ribosome collisions) to the collided ribosomes. A recent report shows that activation of Hel2/ZNF598 by ribosome stalling is independent of GCN2 activation and that these two pathways display different specificity and thresholds for ribosome collisions (55). These data suggest a model in which ribosome collisions trigger the recruitment of independent protein complexes carrying distinct translation initiation repressors such as ZNF598/GIGYF2/4EHP, GCN2/GCN1/LARP1, and recently identified EDF1/GIGYF2/4EHP (49, 56). This would enable the cells to suppress translation of distinct subsets of mRNAs independent of each other. Future studies are required to uncover the importance of each protein complex and to delineate how cells coordinate multiple pathways to respond to different translational stressors.

It is important to emphasize that our data did not rule out other possible mechanisms for GCN2-dependent TOP mRNA translational suppression. These include inhibition of mTORC1 (ATF4-dependent and ATF4-independent) or induction of SGs and P-bodies. It is conceivable that cells use different mechanisms to spatiotemporally regulate the expression of TOP mRNAs.

## Experimental procedures

### CRISPR strategy for 3xFlag tag insertion in frame with GCN1

Alt-R CRISPR-Cas9 system (IDT) was used for endogenously tagging GCN1 in HEK293T cells. N-terminal fusion of 3xFlag tag to one allele of Gcn1 was achieved by delivering Alt-RS.p. Cas9 nuclease protein and Alt-R CRISPR-Cas9 guide RNA (crRNA:tracrRNA duplex) in a ribonucleoprotein (RNP) complex with a single strand DNA HDR donor template (ssODN) according to manufacturer’s instruction. Briefly, RNP assembly was performed by mixing equimolar crRNA and tracrRNA in nuclease-free duplex buffer to a final concentration of 1 μM. The mix was heated at 95 °C for 5 min. Then, 1.5 μl of guide RNA oligos (1 μM) were combined with 1.5 μl diluted Cas9 enzyme (1 μM) and 22 μl of Opti-MEM media and incubated 5 min at room temperature (RT) to produce the RNP complexes. Next, 1 to 3 μM ssODN were added to the RNP complex. Finally, 25 μl of RNP complex mixed with 1.2 μl of Lipofectamine 2000 and 23.8 μl Opti-MEM and incubated for 20 min at RT before transfection of 4000 HEK293T cells. Forty-eight hours post-transfection, single cell dilution of the cells were seeded in 96-well plates to obtain clonal population.

#### HDR donor template (ssODN)

5′GTGGGCCACGCTGTGACCCGGAAGCGTTCCGGAAGCGGTTCCGGAGTCAGCCCCGGCAGGgccgccAccatggactacaaagaccatgacggtgattataaagatcatgacatcgattacaaggatgacgatgacaagGCGGCGGACACGCAGGTGAGGCGGGCGGCTGCGGGGCCAACGCGGCCAGGGACTGGGTGCGGACGGTGGCCGTCG-3′

#### Guide RNA targeting sequence

5′-CGGAGTCAGCCCCGGCAGGA-3′.

### Polysome profiling

#### Cell culture

The polysome profiling protocol employed is previously described (57). Briefly, HEK293T cells were seeded into four 15 cm^2^ culture dishes per condition and maintained in Dulbecco's modified Eagle's medium (DMEM), 10% fetal bovine serum (FBS), penicillin (50 μg/ml), and streptomycin (50 μg/ml) in a 37 °C incubator until they reached 80% confluency on the day of the experiment. DMEM without leucine (226–024; Crystalgen) or control DMEM (226–033; Crystalgen) were combined with 10% dialyzed FBS (A3382001; Gibco) for amino acid deprivation experiments.

#### Lysate preparation and fractionation of polysome

A volume of 200 μl of 10 mg/ml of cycloheximide (CHX) was added to each 15 cm^2^ culture dish and incubated for 5 min in a 37 °C incubator. Then, the cells were gently washed twice with 10 ml ice-cold 1x PBS containing 100 μg/ml CHX. Next, cells were quickly scraped and collected in 5 ml ice-cold 1x PBS (containing CHX) solution and centrifuged at 200*g* for 5 min at 4 °C (Legend XTR Centrifuge, Thermo Scientific). The supernatants were discarded, and the pellets were resuspended in 425 μl of hypotonic buffer (5 mM Tris–HCl [pH 7.5], 1.5 mM KCl, 2.5 mM MgCl2, 1x protease inhibitor solution), 5 μl of 10 mg/ml CHX, 100 units of RNase inhibitor, and 1 μl of 1M DTT. Then, the tubes were vortexed (Vortex -Genie 2, Scientific Industries) for 5 s, and the following reagents were added to the buffer: 25 μl of 10% Triton X-100 (0.5% as the final concentration) and 25 μl of sodium deoxycholate (0.5% as the final concentration) and then vortexed for 5 s. The tubes were then centrifuged at 16,000*g* for 7 min at 4 °C (Eppendorf Centrifuge 5424 R). Next, the supernatants were transferred to 1.5 ml prechilled microtubes. The absorbance was measured at 260 nm (BioDrop) for each sample. About 10% of each lysate was kept at −80 °C to further determine total mRNA. Then, 500 μl of the supernatants were added on the top of sucrose gradient tubes (prepared by Gradient Master, Model 108, BioComp). We established 400 μg as the ideal amount of lysate for our polysome machine to obtain polysome fractions. Samples were centrifuged through the sucrose gradients by ultracentrifugation at 222,228×*g* (36,000 rpm) for 2 h at 4 °C using a SW40Ti rotor (Beckman Coulter, Optima L-90k Ultracentrifuge). Polysome fractionation was performed by the Brandel Gradient Fractionation System (BR-188 Density Gradient Fractionation System). We used the PeakChart data acquisition software (Brandel) for monitoring the polysomes and data acquisition. Trizol (1 ml) was added to each fraction and fractions were frozen immediately in liquid nitrogen and stored in a −80 °C freezer for future RNA extraction.

For ribosome pausing and comigration experiments, cells were treated with ANS (Sigma–Aldrich, A9789) at 1 μg/ml for 15 min in the absence of CHX at 37 °C. Following fractionation, 10% trichloroacetic acid (Fisher bioreagents, catalog no.: #BP555-500) was used to precipitate the proteins from 300 μl of each fraction. After 2 h incubation at 4 °C, the precipitated proteins were centrifuged at 15,000 rpm for 15 min at 4 °C. The pellets were air dried for 15 min and resuspended in 40 μl of 2X SDS-PAGE Laemmli sample buffer (Bio-Rad, catalog no.: # 1610737) and loaded on 8% gels. For amino acid starvation, cells were pretreated with 1 μM ISRIB for 30 min, then washed with PBS two times, and cultured in the amino acid deficient media (DMEM without leucine [226-024; Crystalgen] or methionine [226-025; Crystalgen] combined with 10% dialyzed FBS [A3382001; Gibco]) with 1 μM ISRIB for 1 h. Lysates containing 400 μg of total RNA were treated with RNase A at 0, 0.5, and 5 mg/l for 15 min at RT followed by addition of 200 U of SUPERaseIn. Digested lysates were subjected to centrifugation through 10% to 35% sucrose gradients.

### RT-qPCR

RevertAid RT Reverse Transcription Kit (Thermo Fisher Scientific, catalog no.: #K1691) was used for generation of complementary DNA. iTaq Universal SYBR Green Supermix (Bio-Rad; catalog no.: #1725121) were used for qPCR. The relative expression of genes was normalized to the expression of house-keeping genes including *Gapdh*, *β-Actin*, or 18S rRNA. All (n)s are considered technical replicates unless indicated otherwise.

Primers used for mouse and human RT-qPCR are summarized in Table S1.

### ChIP-qPCR

WT and ATF4 KO MEFs or WT (DR-WT) and GCN2 KO (GCN2-KO-DR) MEFs (ATCC) (∼1X10^7^) were grown to 90% confluence and crosslinked with 1% formaldehyde (methanol-free) at RT for 10 min with gentle agitation. Crosslinking was quenched by the addition of quenching buffer (1M glycine buffer, 125 mM as a final concentration) and incubated at RT for 5 min with gentle agitation. Crosslinked cells were resuspended with lysis buffer, and their nuclei were pelleted and lysed with shearing buffer. All buffers were provided from the truChIP Chromatin shearing kit (520154, Covaris). Chromatin was sonicated with a ultrasonicator to produce fragments from 150 to 1000 bp with enriched fragments between 200 and 500 bp. Fragmented chromatin from approximately 1X10^7^ cell equivalents was incubated overnight at 4 °C with antibody coupled to protein A beads with salmon sperm DNA (16-157, Milipore). Chromatin equivalent to 10% volume used for ChIP was used for input control. Immunoprecipitated complexes were washed sequentially with a low salt wash buffer X 2 (0.1% SDS, 1% Triton X-100, 2 mM EDTA, 20 mM Tris–HCl pH 8.0, 150 mM NaCl), high salt wash buffer X 2 (0.1% SDS, 1% Triton X-100, 2 mM EDTA, 20 mM Tris–HCl pH 8.0, 500 mM NaCl), LiCl wash buffer X 1 (10 mM Tris–HCl pH 8.0, 0.25 M LiCl, 1% NP-40, 1% sodium deoxycholate, 1 mM EDTA), and finally with TE buffer X 2 (pH 8.0). Bead-bound DNA was eluted by addition of 100 μl of elution buffer (1% SDS, 100 mM NaHCO_3_) to the complexes and vortexed slowly for 30 min at RT. Samples were centrifuged for 3 min at 1000*g* and the supernatants were transferred to new tubes. To the tubes, 2 μl of RNase A (10 mg/ml) and 2 μl of proteinase K (20 mg/ml) were added and the tubes were incubated at 65 °C overnight. The next day, the DNA was purified using a commercial kit (D5201, Zymo research, ChIP DNA clean and concentrator). ChIP fold enrichments were normalized to the binding to two nonbound control regions. All (n)s are considered technical replicates unless indicated otherwise.

Primers used for ChIP-qPCR are found in Table S1.

### ChIP-seq

ATF4 ChIP-seq experiments were performed on primary WT and GCN2 KO MEFs ± leucine deprivation for 24 h. For each ChIP, 5 μg of anti-ATF4 (sc-200, Santa-Cruz) was immobilized overnight at 4 °C on 50 μl protein G Dynabeads (catalog no.: #10004D, ThermoFisher) diluted in 500 μl of PBS + 0.5% bovine serum albumin (BSA). The next day, the antibody-conjugated beads were washed twice with PBS + 0.5 % BSA. Sonicated chromatin (75 μg) was diluted in 2.5X ChIP dilution buffer (Tris 20 mM, ph 8, NaCl 100 nM, EDTA 2 mM, pH 8, Triton-X-100 0.5%) + 100 μl of PBS + 0.5% BSA and added to the antibody-bound beads and left to rotate O/N at 4 °C. Then, the beads were washed 3X with 1 ml LiCl wash buffer (Tris 100 nm, LiCl 500 nM, Na-deoxycholate 1%, NP-40 1%), transferred to new tubes, then washed 2X more with LiCl wash buffer followed by a quick wash with TE buffer. DNA was eluted with 300 μl decrosslinking buffer (NaHCO_3_ 0.1 M, SDS 1%) overnight at 65 °C. RNase A (0.2ug/ul final) was added to the samples and left at 37 °C for 1 h following by addition of proteinase K (0.2 μg/μl final) and incubation at 55 °C for 30 min. ChIP DNA was purified using a QIAquick PCR purification kit (Qiagen). Chromatin from two independent experiments each performed with at least five replicates was pooled together prior to library preparation and sequencing.

DNA libraries and sequencing were performed at the Génome Québec Innovation Centre. DNA library preparation was performed using the TruSeq DNA sample preparation kit according to Illumina recommendations. The ChIP DNA libraries were sequenced as single 50 bp reads (tags) using an Ilumina Hiseq 2500 sequencer (Illumina). Raw reads were trimmed for length (n ≥ 50), quality (phred score ≥ 30), and adapter sequence using fastx v0.0.13.2. Trimmed reads were then aligned to the mouse reference genome mm10 using BWA v0.7.12 (58). Peaks were called using MACS v2.1.0 software and default parameters (mfold = [5,50]; false discovery rate [FDR] cutoff = 0.05, --nomodel) using sequenced libraries of either WT or GCN2 KO input DNA as control (59). The regions defined by the peak summit ±150 bp were used for downstream analysis. Peak annotations, tag directory, bed files, and *de novo* motif discovery were performed using HOMER v4.7 (60). For peak intersections, peak list intersections were done using the R package GenomicRanges v1.26.4 (61). Binding peaks were considered overlapping if their peak summits were found within 300 bp or less apart. Binding intensity heatmaps and tag density plots were generated using the R package Genomation v1.9.3 (62) with an estimated fragment length of 200 bp.

### Antibodies

Antibodies used were ATF4 (sc-200) (for ChIP-seq), ATF-4 antibody (D4B8) from Cell Signaling (For ChIP-qPCR), ATF4/CREB-2 (sc-390063, Santa Cruz) (for WB analysis presented in Fig. S3*D*), ATF5 (Santa Cruz, sc-377168), LARP1 (sc-515873, Santa Cruz or ab86359, Abcam), p-eIF2α (ab32157, Abcam), Actin (ab179467, Abcam), GCN1 (ab8613, Abcam), GCN2 (3302S, Cell Signaling), anti-FLAG (F3165, Sigma), antimouse IgG, horeseradish peroxidase conjugated (W402B, Promega), anti-rabbit IgG, horeseradish peroxidase conjugated (W401B, Promega), and G3BP (catalog no.: #611126, BD Transduction Laboratories).

### IP

We generated HEK293T cells that expressed endogenously 3xFlag-tagged GCN1. Chaps cell extract buffer (Cell Signaling Technology #9852) in presence or absence of RNase A (Sigma) was used to lyse the cells. Immunoprecipitation was performed using anti-Flag-M2 affinity gel (Sigma, A2220).

### Functional enrichment analysis

ATF4 ChIP-seq target genes with binding peaks found ± 5 kb of gene transcription start sites in WT and GCN2 KO MEFs ± leucine were used as input for functional enrichment analysis by Ingenuity Pathway Analysis (IPA, www.ingenuity.com), WebGestalt (www.webgestalt.org), and Enrichr (https://maayanlab.cloud/Enrichr/). For IPA analysis, Fisher’s exact test was used to calculate *p*-values determining the probability that the association between genes in the dataset and the canonical pathway is explained by chance alone. Fisher’s exact test *p*-values were corrected for multiple testing (adjusted *p*-value, Padj) using the Benjamini–Hochberg method. KEGG pathway enrichment analysis was performed using WebGestalt. Enrichment of Hallmark signatures (2020) from the Molecular Signatures Database (MSigDB) were determined using Enrichr.

Over-representation of Gene Ontology biological processes from an ATF4-targeted 145 gene subset found bound by ATF4 in MEFs ± leucine within 5 kb of gene transcription start sites was evaluated using WebGestalt. The top 10 significant terms with FDR < 0.05 were determined following a weighted set cover redundancy reduction of functional gene sets comprising a minimum of three genes.

### Lentivirus preparation

About 8 × 10^6^ 293T cells in a 10 cm dish were transfected with 10 μg of Lentivector, 6.5 μg of psPAX2, 3.5 μg of pMD2.G, in presence of 50 μl of Lipofectamine 2000 and 1 ml of Opti-MEM. Twenty-four hours post-transfection, the medium was collected everyday for 48 h and was subjected to centrifugation at 76,000*g* for 1.5 h. The viral pellet was then resuspended in DMEM and 10% heat-inactivated FBS and then rotated overnight at 4 °C. The resulting concentrated virus solution was used to infect cells directly in presence of 8 μg/ml polybrene or was flash frozen in aliquots on dry ice for long term storage at −80 °C.

### Lentivirus plasmids

shRNA against human ATF4 (Sigma; TRCN0000013573)

shRNA against human LARP1 (Sigma; TRCN0000150984)

shRNA against human GCN1 (Sigma; TRCN0000154822)

nontargeting shRNA SHC002 (Sigma)

### Proteomics and data analysis

Samples were run on SDS-PAGE gel and a gel band was subject for in-gel digestion. Gel band was washed in 100 mM ammonium bicarbonate/acetonitrile (ACN) and reduced with 10 mM DTT at 50 °C for 30 min. Cysteines were alkylated with 100 mM iodoacetamide in the dark for 30 min in RT. Gel band was washed in 100 mM ammonium bicarbonate/ACN prior to adding 600 ng trypsin for overnight incubation at 37 °C. Supernatant containing peptides was saved into a new tube. Gel was washed at RT for 10 minutes with gentle shaking in 50% ACN/5% formic acid (FA), and supernatant was saved to peptide solution. Wash step was repeated each by 80% ACN/5% FA and 100% ACN, and all supernatant was saved, then subject to the speedvac dry. After lyophilization, peptides were reconstituted with 5% ACN/0.1% FA in water and injected onto a trap column (150 μm ID X 3 cm in-house packed with ReproSil C18, 3 μm) coupled with an analytical column (75 μm ID X 10.5 cm, PicoChip column packed with ReproSil C18, 3 μm) (New Objectives, Inc). Samples were separated using a linear gradient of solvent A (0.1% FA in water) and solvent B (0.1% FA in ACN) over 120 min using a Dionex UltiMate 3000 Rapid Separation nanoLC (ThermoFisher Scientific). MS data were obtained on a Orbitrap Elite Mass Spectrometer (Thermo Fisher Scientific Inc). Data were searched using Mascot (Matrix Science) v.2.5.1 against the Swiss-Prot Human database (2019), and results were reported at 1% FDR in Scaffold v.4.8.4 (Proteome Software).

The GCN1 AP-MS data was analyzed by SAINT (63) using REPRINT (64) with default parameters. A purification done from HEK293T cells not expressing 3xFlag-tagged GCN1 was used as a negative control for the SAINT analysis. The dot plot displaying the GCN1 interactors with a SAINT score ≥0.9 was generated using the ProHits-viz suite (65). For the dot plot generator, SAINT scores were used for filtering, and total spectral counts were used for displaying abundance with the following parameters: Primary filter = SAINT score of 1; Secondary filter = SAINT score of 0.9; Minimum abundance value = 0; Maximum abundance value = 40.

### Ribosome profiling analysis

Trimming and alignment were performed as described in GSE113751. The R package DESeq2 was used to calculate footprints per million reads for each Gencode v24 gene and normalize across each sample, for the six HEK293T samples in GSE113751 (GSM3118956-61).

## Data availability

ChIP-seq data are available in the Gene Expression Omnibus (GEO) database under the accession number GSE166590.

## Supporting information

This article contains supporting information

## Conflict of interest

The authors declare that they have no conflicts of interest with the contents of this article.

## Supporting information

Separate State file

Supplemental Information titles and legends

Table S2

New Figure S1

New Figure S2

New Figure S3

New Figure S4
