# Supplementary material for: The amino acid sensor GCN2 suppresses terminal oligopyrimidine (TOP) mRNA translation via La-related protein 1 (LARP1)
Source: J Biol Chem. 2022 Jul 19;298(9):102277. doi: 10.1016/j.jbc.2022.102277 (PMC9396407; doi:10.1016/j.jbc.2022.102277)
Supplement: Separate State file [file mmc1.docx]

The graphs that are presented here are the reuse of exact graphs appeared in the main figures. Panel number corresponding to the main figures are indicated above each graph.

**Figure 2C**

Within IP samples

| Bonferroni's multiple comparisons test | Mean Diff. | 95.00% CI of diff. | Below threshold? | Summary |
| --- | --- | --- | --- | --- |
|  |  |  |  |  |
| WT - KO |  |  |  |  |
| +Leu | 0.005580 | -0.1309 to 0.1421 | No | ns |
| -Leu | 0.2376 | 0.1011 to 0.3741 | Yes | ** |

| Bonferroni's multiple comparisons test | Mean Diff. | 95.00% CI of diff. | Below threshold? | Summary |
| --- | --- | --- | --- | --- |
|  |  |  |  |  |
| +Leu - -Leu |  |  |  |  |
| WT | -0.4948 | -0.6313 to -0.3583 | Yes | **** |
| KO | -0.2628 | -0.3993 to -0.1263 | Yes | *** |

**Figure 2C**

Within IP samples

| Bonferroni's multiple comparisons test | Mean Diff. | 95.00% CI of diff. | Below threshold? | Summary |
| --- | --- | --- | --- | --- |
|  |  |  |  |  |
| WT - KO |  |  |  |  |
| +Leu | -0.04157 | -0.1747 to 0.09159 | No | ns |
| -Leu | 0.3516 | 0.2185 to 0.4848 | Yes | **** |

| Bonferroni's multiple comparisons test | Mean Diff. | 95.00% CI of diff. | Below threshold? | Summary |
| --- | --- | --- | --- | --- |
|  |  |  |  |  |
| +Leu - -Leu |  |  |  |  |
| WT | -0.5358 | -0.6689 to -0.4026 | Yes | **** |
| KO | -0.1426 | -0.2757 to -0.009431 | Yes | * |

**Figure 2C**

Within IP samples

| Bonferroni's multiple comparisons test | Mean Diff. | 95.00% CI of diff. | Below threshold? | Summary |
| --- | --- | --- | --- | --- |
|  |  |  |  |  |
| WT - KO |  |  |  |  |
| +Leu | -0.002916 | -0.2017 to 0.1959 | No | ns |
| -Leu | 0.8952 | 0.6923 to 1.098 | Yes | **** |

| Bonferroni's multiple comparisons test | Mean Diff. | 95.00% CI of diff. | Below threshold? | Summary |
| --- | --- | --- | --- | --- |
|  |  |  |  |  |
| +Leu - -Leu |  |  |  |  |
| WT | -1.112 | -1.311 to -0.9136 | Yes | **** |
| KO | -0.2143 | -0.4131 to -0.01550 | Yes | * |

**Figure 2C**

Within IP samples

| Bonferroni's multiple comparisons test | Mean Diff. | 95.00% CI of diff. | Below threshold? | Summary |
| --- | --- | --- | --- | --- |
|  |  |  |  |  |
| WT - KO |  |  |  |  |
| +Leu | 0.03320 | -0.1157 to 0.1821 | No | ns |
| -Leu | 0.5369 | 0.3880 to 0.6858 | Yes | **** |

| Bonferroni's multiple comparisons test | Mean Diff. | 95.00% CI of diff. | Below threshold? | Summary |
| --- | --- | --- | --- | --- |
|  |  |  |  |  |
| +Leu - -Leu |  |  |  |  |
| WT | -0.7203 | -0.8692 to -0.5714 | Yes | **** |
| KO | -0.2166 | -0.3655 to -0.06768 | Yes | ** |

**Figure 2D**

| Bonferroni's multiple comparisons test | Mean Diff. | 95.00% CI of diff. | Below threshold? | Summary |
| --- | --- | --- | --- | --- |
|  |  |  |  |  |
| WT - KO |  |  |  |  |
| +Leu | 0.0009680 | -0.1562 to 0.1581 | No | ns |
| -Leu | 0.3803 | 0.2232 to 0.5375 | Yes | *** |

| Bonferroni's multiple comparisons test | Mean Diff. | 95.00% CI of diff. | Below threshold? | Summary |
| --- | --- | --- | --- | --- |
|  |  |  |  |  |
| +Leu - -Leu |  |  |  |  |
| WT | -0.5155 | -0.6727 to -0.3584 | Yes | **** |
| KO | -0.1362 | -0.2933 to 0.02100 | No | ns |

**Figure 2D**

| Bonferroni's multiple comparisons test | Mean Diff. | 95.00% CI of diff. | Below threshold? | Summary |
| --- | --- | --- | --- | --- |
|  |  |  |  |  |
| WT - KO |  |  |  |  |
| +Leu | -0.004172 | -0.5798 to 0.5715 | No | ns |
| -Leu | 1.133 | 0.5569 to 1.708 | Yes | *** |

| Bonferroni's multiple comparisons test | Mean Diff. | 95.00% CI of diff. | Below threshold? | Summary |
| --- | --- | --- | --- | --- |
|  |  |  |  |  |
| +Leu - -Leu |  |  |  |  |
| WT | -1.233 | -1.808 to -0.6569 | Yes | *** |
| KO | -0.09575 | -0.6714 to 0.4799 | No | ns |

**Figure 2D**

| Bonferroni's multiple comparisons test | Mean Diff. | 95.00% CI of diff. | Below threshold? | Summary |
| --- | --- | --- | --- | --- |
|  |  |  |  |  |
| WT - KO |  |  |  |  |
| +Leu | -0.02670 | -0.5705 to 0.5172 | No | ns |
| -Leu | 0.8562 | 0.3123 to 1.400 | Yes | ** |

| Bonferroni's multiple comparisons test | Mean Diff. | 95.00% CI of diff. | Below threshold? | Summary |
| --- | --- | --- | --- | --- |
|  |  |  |  |  |
| +Leu - -Leu |  |  |  |  |
| WT | -1.976 | -2.520 to -1.432 | Yes | **** |
| KO | -1.093 | -1.637 to -0.5496 | Yes | *** |

**Figure 2D**

| Bonferroni's multiple comparisons test | Mean Diff. | 95.00% CI of diff. | Below threshold? | Summary |
| --- | --- | --- | --- | --- |
|  |  |  |  |  |
| WT - KO |  |  |  |  |
| +Leu | -0.04506 | -1.807 to 1.717 | No | ns |
| -Leu | 2.704 | 0.9413 to 4.466 | Yes | ** |

| Bonferroni's multiple comparisons test | Mean Diff. | 95.00% CI of diff. | Below threshold? | Summary |
| --- | --- | --- | --- | --- |
|  |  |  |  |  |
| +Leu - -Leu |  |  |  |  |
| WT | -7.083 | -8.845 to -5.320 | Yes | **** |
| KO | -4.334 | -6.096 to -2.572 | Yes | *** |

**Figure 2E**

Within IP samples

| Bonferroni's multiple comparisons test | Mean Diff. | 95.00% CI of diff. | Below threshold? | Summary |
| --- | --- | --- | --- | --- |
|  |  |  |  |  |
| WT - KO |  |  |  |  |
| +Leu | 0.06100 | -0.02457 to 0.1466 | No | ns |
| -Leu | 0.5967 | 0.5111 to 0.6822 | Yes | **** |

| Bonferroni's multiple comparisons test | Mean Diff. | 95.00% CI of diff. | Below threshold? | Summary |
| --- | --- | --- | --- | --- |
|  |  |  |  |  |
| +Leu - -Leu |  |  |  |  |
| WT | -0.5618 | -0.6473 to -0.4762 | Yes | **** |
| KO | -0.02610 | -0.1117 to 0.05947 | No | ns |

**Figure 2E**

Within IP samples

| Bonferroni's multiple comparisons test | Mean Diff. | 95.00% CI of diff. | Below threshold? | Summary |
| --- | --- | --- | --- | --- |
|  |  |  |  |  |
| WT - KO |  |  |  |  |
| +Leu | 0.04041 | -0.001231 to 0.08206 | No | ns |
| -Leu | 0.3301 | 0.2885 to 0.3718 | Yes | **** |

| Bonferroni's multiple comparisons test | Mean Diff. | 95.00% CI of diff. | Below threshold? | Summary |
| --- | --- | --- | --- | --- |
|  |  |  |  |  |
| +Leu - -Leu |  |  |  |  |
| WT | -0.3465 | -0.3881 to -0.3049 | Yes | **** |
| KO | -0.05677 | -0.09842 to -0.01513 | Yes | ** |

**Figure 2E**

Within IP samples

| Bonferroni's multiple comparisons test | Mean Diff. | 95.00% CI of diff. | Below threshold? | Summary |
| --- | --- | --- | --- | --- |
|  |  |  |  |  |
| WT - KO |  |  |  |  |
| +Leu | -0.01524 | -0.3169 to 0.2864 | No | ns |
| -Leu | 1.082 | 0.7803 to 1.384 | Yes | **** |

| Bonferroni's multiple comparisons test | Mean Diff. | 95.00% CI of diff. | Below threshold? | Summary |
| --- | --- | --- | --- | --- |
|  |  |  |  |  |
| +Leu - -Leu |  |  |  |  |
| WT | -1.019 | -1.320 to -0.7170 | Yes | **** |
| KO | 0.07849 | -0.2232 to 0.3802 | No | ns |

**Figure 2E**

Within IP samples

| Bonferroni's multiple comparisons test | Mean Diff. | 95.00% CI of diff. | Below threshold? | Summary |
| --- | --- | --- | --- | --- |
|  |  |  |  |  |
| WT - KO |  |  |  |  |
| +Leu | -0.05737 | -0.2218 to 0.1070 | No | ns |
| -Leu | 0.6093 | 0.4449 to 0.7737 | Yes | **** |

| Bonferroni's multiple comparisons test | Mean Diff. | 95.00% CI of diff. | Below threshold? | Summary |
| --- | --- | --- | --- | --- |
|  |  |  |  |  |
| +Leu - -Leu |  |  |  |  |
| WT | -0.5708 | -0.7352 to -0.4064 | Yes | **** |
| KO | 0.09586 | -0.06856 to 0.2603 | No | ns |

**Figure 2F**

| Bonferroni's multiple comparisons test | Mean Diff. | 95.00% CI of diff. | Below threshold? | Summary |
| --- | --- | --- | --- | --- |
|  |  |  |  |  |
| WT - KO |  |  |  |  |
| +Leu | 0.02570 | -0.5979 to 0.6493 | No | ns |
| -Leu | 0.4287 | -0.1949 to 1.052 | No | ns |

| Bonferroni's multiple comparisons test | Mean Diff. | 95.00% CI of diff. | Below threshold? | Summary |
| --- | --- | --- | --- | --- |
|  |  |  |  |  |
| +Leu - -Leu |  |  |  |  |
| WT | -1.009 | -1.633 to -0.3857 | Yes | ** |
| KO | -0.6064 | -1.230 to 0.01724 | No | ns |

**Figure 2F**

| Bonferroni's multiple comparisons test | Mean Diff. | 95.00% CI of diff. | Below threshold? | Summary |
| --- | --- | --- | --- | --- |
|  |  |  |  |  |
| WT - KO |  |  |  |  |
| +Leu | 0.01194 | -2.905 to 2.928 | No | ns |
| -Leu | 12.00 | 9.082 to 14.92 | Yes | **** |

| Bonferroni's multiple comparisons test | Mean Diff. | 95.00% CI of diff. | Below threshold? | Summary |
| --- | --- | --- | --- | --- |
|  |  |  |  |  |
| +Leu - -Leu |  |  |  |  |
| WT | -16.73 | -19.64 to -13.81 | Yes | **** |
| KO | -4.740 | -7.657 to -1.824 | Yes | ** |

**Figure 2F**

| Bonferroni's multiple comparisons test | Mean Diff. | 95.00% CI of diff. | Below threshold? | Summary |
| --- | --- | --- | --- | --- |
|  |  |  |  |  |
| WT - KO |  |  |  |  |
| +Leu | -0.003856 | -0.9570 to 0.9493 | No | ns |
| -Leu | 2.282 | 1.329 to 3.235 | Yes | *** |

| Bonferroni's multiple comparisons test | Mean Diff. | 95.00% CI of diff. | Below threshold? | Summary |
| --- | --- | --- | --- | --- |
|  |  |  |  |  |
| +Leu - -Leu |  |  |  |  |
| WT | -2.710 | -3.663 to -1.756 | Yes | **** |
| KO | -0.4238 | -1.377 to 0.5294 | No | ns |

**Figure 2F**

| Bonferroni's multiple comparisons test | Mean Diff. | 95.00% CI of diff. | Below threshold? | Summary |
| --- | --- | --- | --- | --- |
|  |  |  |  |  |
| WT - KO |  |  |  |  |
| +Leu | -0.01721 | -2.647 to 2.612 | No | ns |
| -Leu | 13.84 | 11.21 to 16.47 | Yes | **** |

| Bonferroni's multiple comparisons test | Mean Diff. | 95.00% CI of diff. | Below threshold? | Summary |
| --- | --- | --- | --- | --- |
|  |  |  |  |  |
| +Leu - -Leu |  |  |  |  |
| WT | -20.45 | -23.08 to -17.82 | Yes | **** |
| KO | -6.591 | -9.221 to -3.962 | Yes | *** |

**Figure 3C**


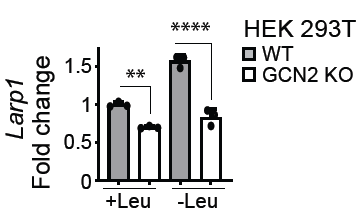


| Bonferroni's multiple comparisons test | Mean Diff. | 95.00% CI of diff. | Below threshold? | Summary |
| --- | --- | --- | --- | --- |
|  |  |  |  |  |
| KO - WT |  |  |  |  |
| +Leu | -0.2997 | -0.4545 to -0.1450 | Yes | ** |
| -Leu | -0.7432 | -0.8980 to -0.5884 | Yes | **** |

| Bonferroni's multiple comparisons test | Mean Diff. | 95.00% CI of diff. | Below threshold? | Summary |
| --- | --- | --- | --- | --- |
|  |  |  |  |  |
| -Leu - +Leu |  |  |  |  |
| WT | 0.5787 | 0.4240 to 0.7335 | Yes | **** |
| KO | 0.1353 | -0.01951 to 0.2900 | No | ns |

**Figure 3C**


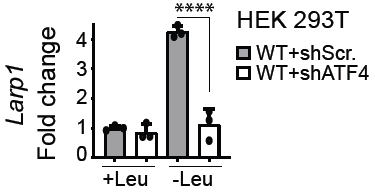


| Bonferroni's multiple comparisons test | Mean Diff. | 95.00% CI of diff. | Below threshold? | Summary |
| --- | --- | --- | --- | --- |
|  |  |  |  |  |
| shATF4 - sch |  |  |  |  |
| +Leu | -0.1339 | -0.8207 to 0.5530 | No | ns |
| -Leu | -3.115 | -3.802 to -2.428 | Yes | **** |

| Bonferroni's multiple comparisons test | Mean Diff. | 95.00% CI of diff. | Below threshold? | Summary |
| --- | --- | --- | --- | --- |
|  |  |  |  |  |
| -Leu - +Leu |  |  |  |  |
| sch | 3.250 | 2.563 to 3.937 | Yes | **** |
| shATF4 | 0.2689 | -0.4180 to 0.9557 | No | ns |
