# Supplementary material for: The amino acid sensor GCN2 suppresses terminal oligopyrimidine (TOP) mRNA translation via La-related protein 1 (LARP1)
Source: J Biol Chem. 2022 Jul 19;298(9):102277. doi: 10.1016/j.jbc.2022.102277 (PMC9396407; doi:10.1016/j.jbc.2022.102277)
Supplement: Supplemental Information titles and legends [file mmc2.docx]

**Supplemental Information titles and legends**

**Figure S1:** **ATF4 ChIP-seq analysis in WT and GCN2 KO MEFs**

(A) De novo motifs (top 3) enriched in ATF4 ChIP-seq binding events in WT and GCN2 KO MEFs in the presence or absence of Leucine (Leu) for 24h. ChIP-seq data represent a single sequencing experiment on a ChIP conducted using chromatin pooled from 2 independent experiments each performed with at least 5 replicates.

(B) ATF4 ChIP-seq location analysis (top) and the number of peaks relative to gene TSSs (bottom) from WT and GCN2 KO MEFs in the presence or absence of Leucine (Leu) for 24h.

(C) The top enriched IPA canonical pathway of ATF4 target genes involved in tRNA charging with binding peaks ± 5kb of gene TSSs. Enrichments were increased upon Leucine (Leu) deprivation and decreased by loss of GCN2.

(D) UCSC Genome browser views for ATF4 ChIP-seq binding events in WT and GCN2 KO MEFs that have been exposed to control (+Leu) or leucine deficient (-Leu) medium for 24h at a subset of ATF4 known targets. ATF4 ChIP-seq binding profiles in WT and ATF4 KO MEFs (control) treated with tunicamycin (Tm) (GSE35681)^41^ are also shown below each panel validating the specificity of the ATF4 targets found.

(E) Enriched (adjusted P-value < 0.05) MSigDB Hallmark 2020 gene signatures and associated genes identified using the Enrich r program with ATF4 ChIP-seq target gene sets with binding peaks found within ± 5kb of gene TSSs.

**Figure S2: GCN2 controls expression of *LARP1* and TOP mRNA translation.**

(A) Western blot analysis of LARP1 in WT (DR-Wildtype) and GCN2 KO (GCN2-KO-DR) MEFs (ATCC) cultured in the absence of Leucine (-Leu) for 0 h, 6 h or 24 h. SE; Short exposure, LE; Long exposure.

(B) Log2 fold-changes in FPM between the GCN2 KO and WT cells described in Fig. 3A for the 5'TOP mRNAs (n=1).

**Figure S3: GCN2 control TOP mRNA translation via regulating LARP1 expression**

(A and B) Assessment of global protein synthesis by puromycin incorporation assay in WT and GCN2 KO cells in presence (A) or absence (B) of L-leucine for 6 h. Data are presented as means ± SD (n=3), Unpaired t test. * p<0.05 and *** p < 0.001.

(C) Polysomal distribution of the indicated TOP mRNAs in cells exposed to L-leucine deficient (-Leu) medium for 6h as determined by RT-qPCR. Values are expressed as a percentage of total levels of corresponding mRNAs in all fractions. P, Pre: fractions 1-4; L, Light: fractions 5-10 ; H, Heavy: fractions 11-13 as indicated in Figure 3E. Data are presented as means ± SD (n=3). * p < 0.05, ** p < 0.01, and *** p < 0.001; Two-way ANOVA followed by Bonferroni post-hoc test.

(D) Immunoblot analysis of WT and GCN2 KO HEK 293T cells in the presence of scrambled shRNA (Scr.) or shRNA against *LARP1*. LARP1 KO cells are shown as a control.

(E) Absorption profiles of ribosomes from WT HEK293T cells in the presence of scrambled shRNA (Scr.) or shRNA against LARP1 (shLARP1). RT-qPCR analysis of *RPS6* and *RPS20* in pooled polysome fractions of cells exposed to L-leucine deficient (-Leu) medium for 6h is shown below the profiles. P, Pre: fractions 1-4; L, Light: fractions 5-10 ; H, Heavy: fractions 11-13. (n=2). * p < 0.05, ** p < 0.01, and *** p < 0.001; Two-way ANOVA followed by Bonferroni post-hoc test.

(F) Absorption profiles of ribosomes from GCN2 KO (KO) HEK293T cells in the presence of scrambled shRNA (Scr.) or shRNA against LARP1 (shLARP1). RT-qPCR analysis of *RPS6* and *RPS20* in pooled polysome fractions of cells exposed to L-leucine deficient (-Leu) medium for 6h is shown below the profiles. P, Pre: fractions 1-4; L, Light: fractions 5-10 ; H, Heavy: fractions 11-13. Data are presented as means ± SD (n=3). * p < 0.05, ** p < 0.01, and *** p < 0.001; Two-way ANOVA followed by Bonferroni post-hoc test.

**Figure S4: Anisomycin treatment promotes LARP1 and GCN1 interaction**

(A) Absorption profiles of ribosomes from DMSO and ANS (1μg/ml) treated HEK 293T cells for 15 min. 40S and 60S denote the corresponding ribosomal subunits and 80S refers to monosomes.

(B) Immunoblot analysis of WT HEK293T cells that were exposed to DMSO or anisomycin (ANS) for 15 minutes and cytosol was fractionated with sucrose density gradients. Precipitated proteins from the polysome fractions (fractions 6-12) were examined by western blotting.

(C-D) Immunoblots and quantification of immunoprecipitates prepared from HEK293T cells endogenously expressing 3xFlag-tagged GCN1 in the presence or absence of anisomycin (ANS) at 1μg/ml for 30 minutes. Data are presented as means ± SD (n=3) Paired t test. * p<0.05.
