# Supplementary material for: The amino acid sensor GCN2 suppresses terminal oligopyrimidine (TOP) mRNA translation via La-related protein 1 (LARP1)
Source: J Biol Chem. 2022 Jul 19;298(9):102277. doi: 10.1016/j.jbc.2022.102277 (PMC9396407; doi:10.1016/j.jbc.2022.102277)
Supplement: New Figure S1 [file mmc5.pdf]

Figure S1

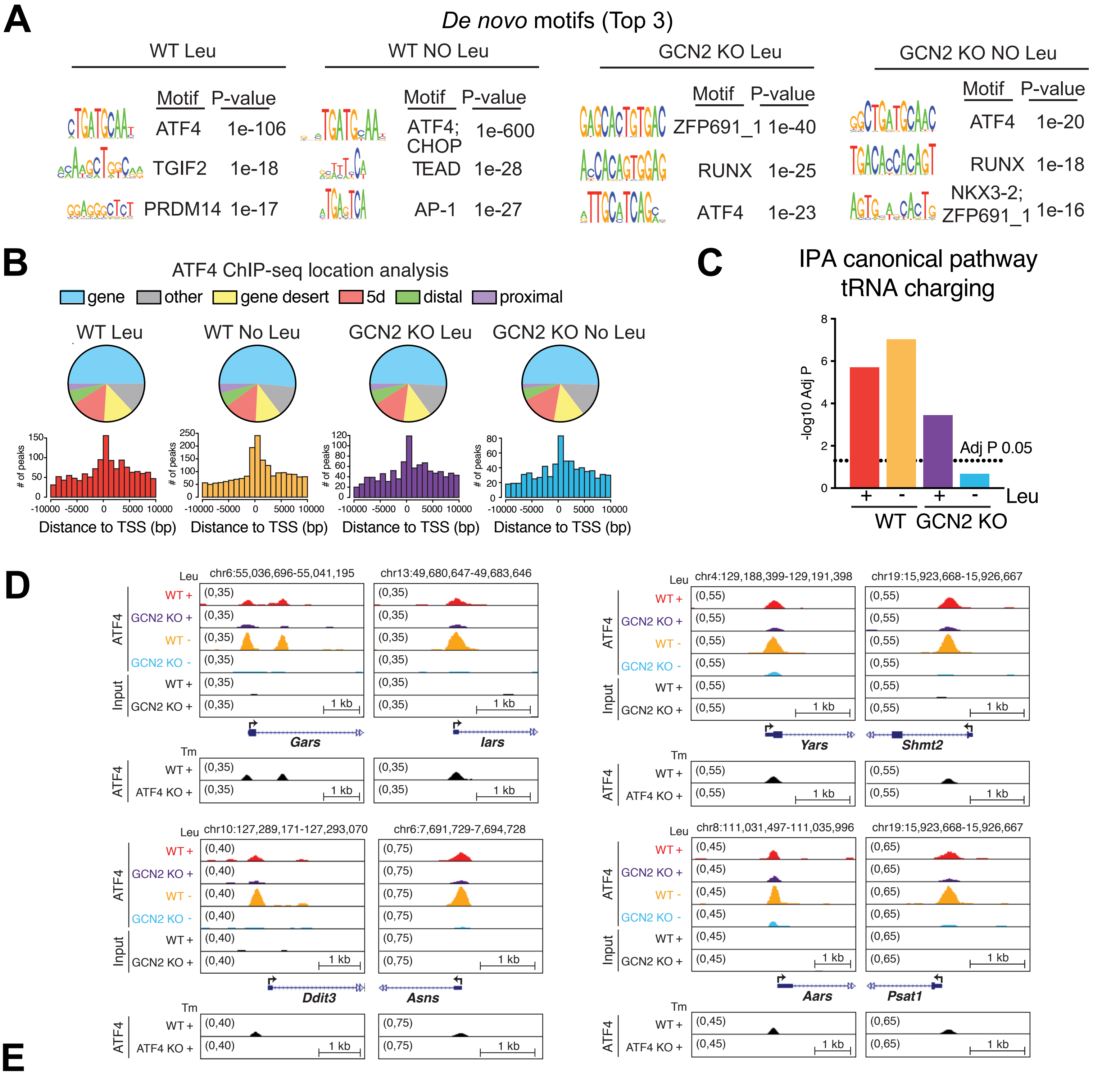

E

MSigDB Hallmark 2020	Overlap	Adj. Pvalue	Genes
**WT Leu (776)**			
Unfolded Protein Response	15/113	1.56E-03	HSPA9;CEBPB;CEBPG;ASNS;SLC1A4;RRP9;HERPUD1;VEGFA;XPOT;MTHFD2;PSAT1;DDIT4;EIF4EBP1;BANF1;CHAC1
mTORC1 Signaling	18/200	2.06E-02	HSPA9;SHMT2;ASNS;SLC1A4;VLDLR;RRP9;PSMC6;NFIL3;MTHFD2;PSAT1;DDIT3;DDIT4;TRIB3;PHGDH;NUPR1;ALDOA;EEF1E1;BCAT1
**WT No Leu (949)**			
Unfolded Protein Response	22/113	7.10E-07	TSPYL2;HSPA9;HSPA5;WFS1;CEBPG;ASNS;SLC1A4;HERPUD1;VEGFA;XPOT;MTHFD2;PSAT1;STC2;DDIT4;EIF4EBP1;CCL2;BANF1;CHAC1;ALDH18A1;ATF6;ATF3;PAIP1
mTORC1 Signaling	29/200	2.03E-06	PPP1R15A;SHMT2;SLC2A1;SLC1A4;GLRX;VLDLR;SLC7A11;CORO1A;ACACA;NFIL3;PHGDH;HSPA9;HSPA5;GOT1;GSR;ASNS;EIF2S2;PFKL;PSMC6;MTHFD2;PSAT1;DDIT3;CTH;DDIT4;TCEA1;TRIB3;NUPR1;BCAT1;SQSTM1
Hypoxia	23/200	1.34E-03	CDKN1C;PPP1R15A;HSPA5;GAA;CAV1;PDGFB;SLC2A1;GLRX;VLDLR;F3;ETS1;NDRG1;PGF;VEGFA;GRHPR;PFKL;NFIL3;DDIT3;STC2;DDIT4;B4GALNT2;FBP1;ATF3
Allograft Rejection	20/200	1.72E-02	IL11;CD74;CCL22;INHBB;CARTPT;F2;ETS1;RPL3L;IL2;TLR1;FGR;CD8A;STAB1;ST8SIA4;STAT4;UBE2N;FLNA;CCL2;BCAT1;EIF3A
**GCN2 KO Leu (577)**			
N/A			
**GCN2 KO No Leu (413)**			
N/A			
