# Supplementary figures and images for: The amino acid sensor GCN2 suppresses terminal oligopyrimidine (TOP) mRNA translation via La-related protein 1 (LARP1)
Source: J Biol Chem. 2022 Jul 19;298(9):102277. doi: 10.1016/j.jbc.2022.102277 (PMC9396407; doi:10.1016/j.jbc.2022.102277)

Figure S2

A

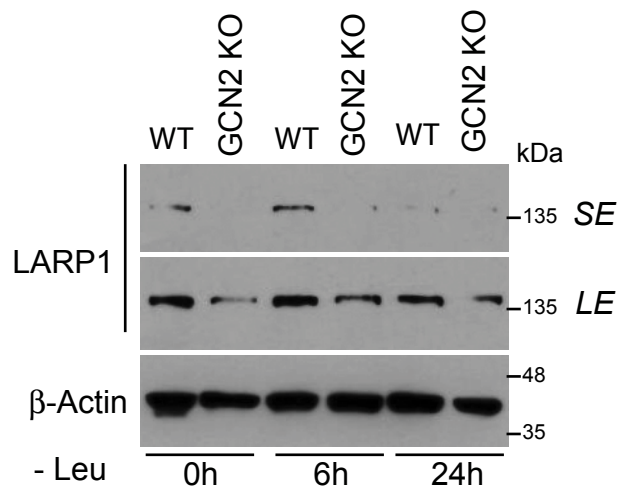

B

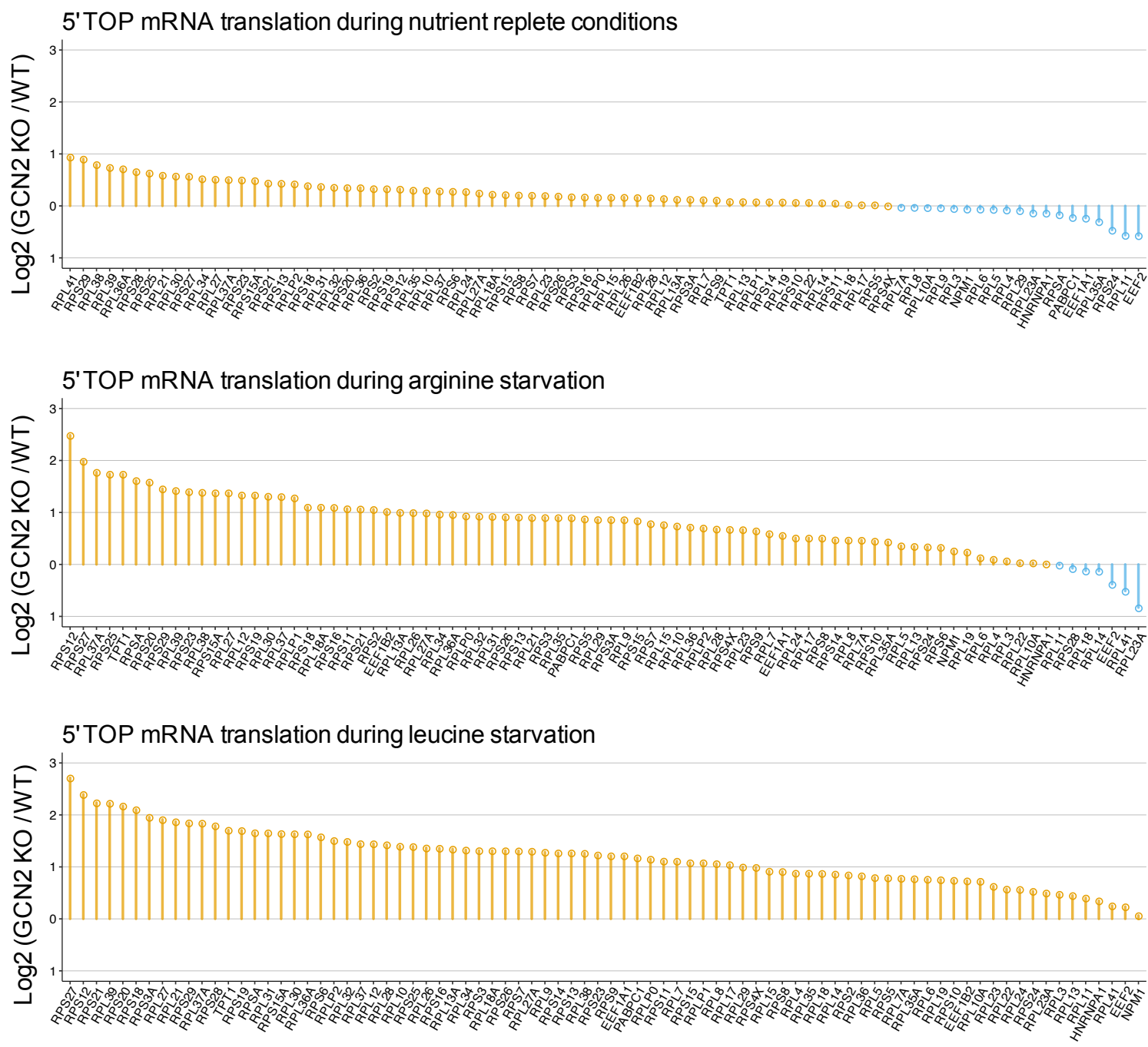

Supplement: New Figure S2 [file mmc6.pdf]

# Figure S3

## A

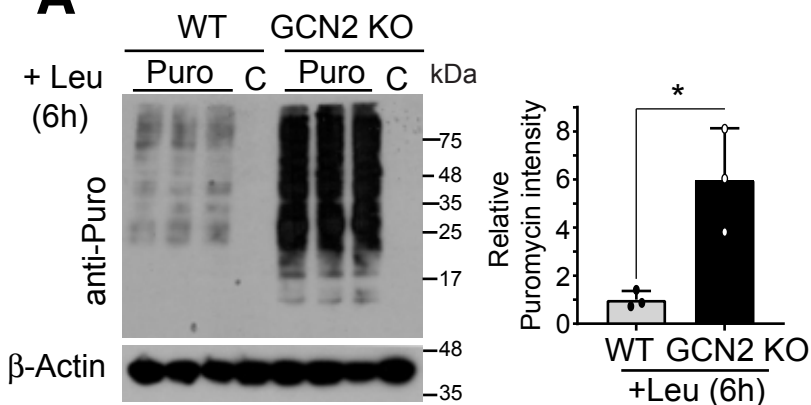

## B

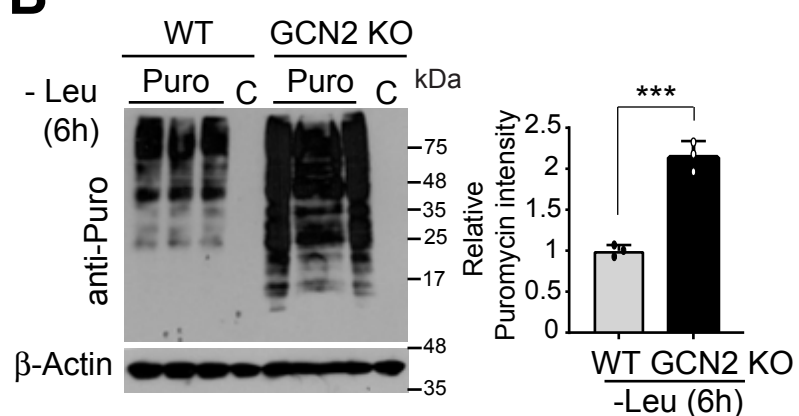

## C

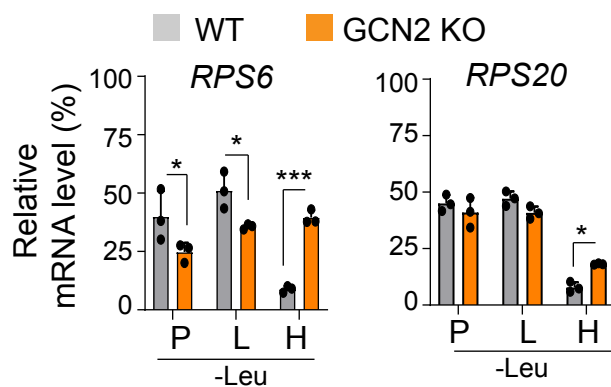

## D

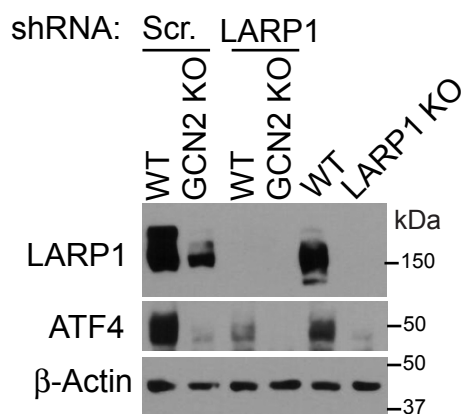

## E

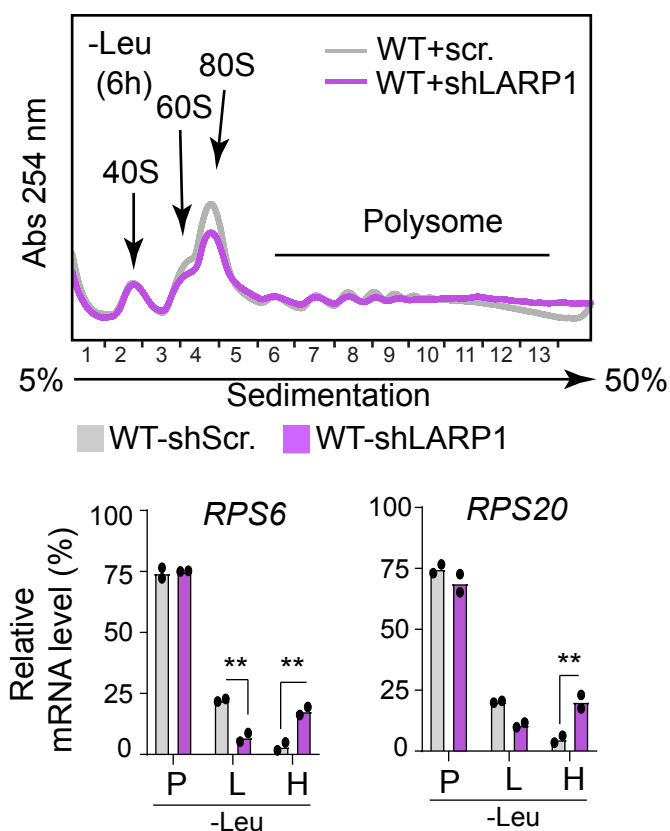

## F

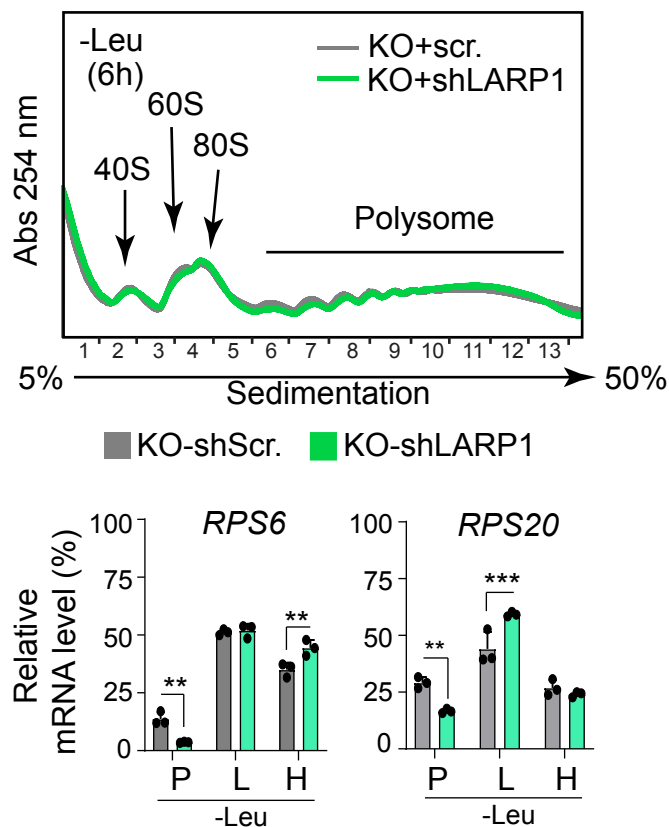

Supplement: New Figure S3 [file mmc7.pdf]

Figure S4

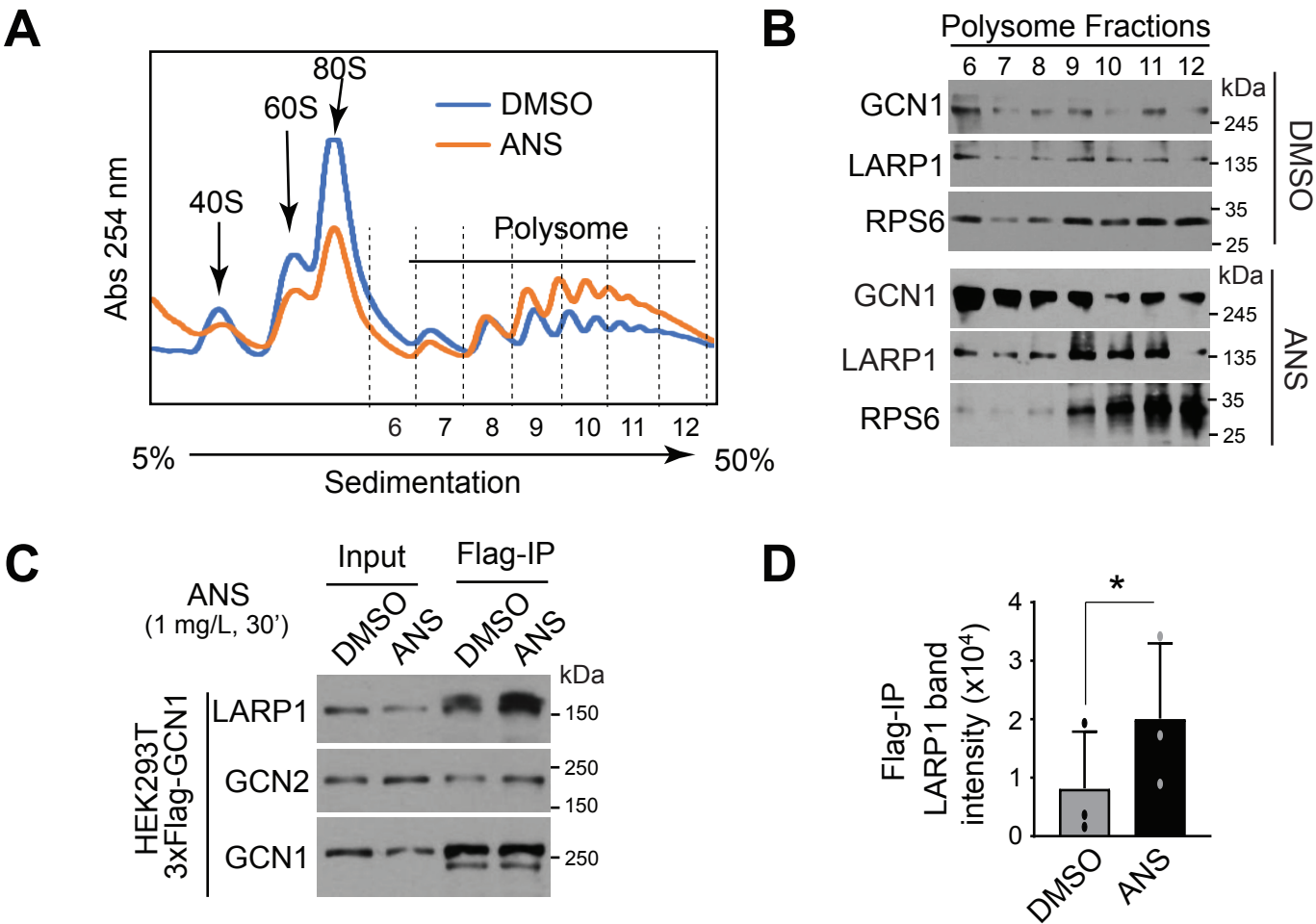

Supplement: New Figure S4 [file mmc8.pdf]
